# Seasonal Patterns and Subtype Distribution of Influenza Viruses in a Tertiary Care Hospital in North India: A One-Year Observational Study

**DOI:** 10.7759/cureus.94428

**Published:** 2025-10-12

**Authors:** Neha Sharma, Shiv Prakash Sharma

**Affiliations:** 1 Microbiology, Bharatpur Medical College, Bharatpur, IND; 2 Preventive Medicine, Rajasthan University of Health Sciences - College of Medical Sciences, Jaipur, IND

**Keywords:** epidemiology, india, influenza, monsoon, rrt-pcr, seasonality, surveillance, tertiary care center

## Abstract

Introduction: Influenza is a major global cause of morbidity and mortality, with complex seasonality that varies by geography. Data from tropical regions like India are limited but essential for guiding vaccination and preparedness strategies. This study examined seasonal patterns, subtype distribution, and demographic characteristics of influenza virus infections in North India.

Methods: A laboratory-based observational study was conducted at a tertiary hospital in Jaipur from July 2019 to June 2020. Throat swabs from 7,231 patients with influenza-like illness (ILI) were tested for influenza A and B viruses using real-time reverse transcription-polymerase chain reaction (rRT-PCR). Demographic and clinical data were analyzed using descriptive statistics and chi-square tests.

Results: Of the 7,231 samples tested, 598 were positive for influenza (8.26%). Influenza A was predominant, 500 cases (83.61%), compared to influenza B, 98 cases (16.39%). A distinct bimodal seasonality was observed, with peaks during the late monsoon (August-September 2019; positivity rates of 110/364 (30.22%) and 193/838 (23.03%), respectively) and late winter (March 2020; 216/1536 (14.06%)). No activity was detected from April to June 2020. The highest burden was among adults aged 20-40 years (233/598; 38.96%), with a significant male predominance (349/598; 58.4%; p<0.0001). Hospitalization was required for 223 cases (37.3%).

Conclusion: Influenza in North India follows a biannual pattern, with peaks in the monsoon and winter. The high burden in young adults underscores their role in transmission. These findings emphasize the need for region-specific vaccination timing and continuous surveillance to strengthen public health preparedness.

## Introduction

Acute respiratory tract infections represent a significant global public health burden, with influenza viruses being a predominant causative agent of seasonal epidemics [1]. Characterized by high mutability and transmissibility, influenza viruses cause a spectrum of illness, from mild afebrile infection to severe pneumonia and death, particularly among high-risk groups such as the young, the elderly, and those with comorbid conditions [2]. The World Health Organization (WHO) estimates that annual influenza epidemics result in three to five million cases of severe illness and 290,000 to 650,000 respiratory deaths globally each year [3].

Influenza viruses are classified into types A, B, C, and D. Influenza A and B are the primary types responsible for seasonal outbreaks in humans [4]. Influenza A viruses are further subtyped based on their surface glycoproteins, hemagglutinin (H) and neuraminidase (N), with A(H1N1)pdm09 and A(H3N2) being the predominant subtypes currently circulating in humans. Influenza B viruses are divided into two lineages: B/Victoria and B/Yamagata [5]. The constant antigenic evolution of these viruses through antigenic drift and, for influenza A, antigenic shift necessitates continuous surveillance to monitor circulating strains [6].

The epidemiology of influenza is notably influenced by seasonal patterns, which vary considerably across different geographic and climatic zones. In temperate regions, influenza activity typically peaks during the winter months [7]. In contrast, tropical countries like India experience more complex transmission patterns, often with biannual peaks or extended periods of circulation influenced by monsoon seasons and varying temperatures [8]. This variability presents a unique challenge for formulating effective public health responses, including the timing of vaccination campaigns and the implementation of non-pharmaceutical interventions (NPIs) [9].

India, with its diverse climatic conditions, lacks a unified influenza seasonality pattern. Data from various regions have shown peaks coinciding with winter, monsoon, or post-monsoon periods [10]. Therefore, regional data are critical for understanding local transmission dynamics. Continuous virological surveillance is the cornerstone for tracking these trends, identifying predominant subtypes, detecting the emergence of novel strains, and providing evidence to inform the selection of vaccine strains by national and international health agencies [11].

This study was undertaken to describe the seasonal patterns, subtype distribution, and demographic characteristics of influenza viruses detected at a tertiary care hospital in Jaipur, North India, over a one-year period.

## Materials and methods

Study design and setting

A laboratory-based, observational, descriptive study was conducted over 12 months, from July 2019 to June 2020. The study was carried out at the Advanced Research Laboratory of the Department of Microbiology, a DHR-established Viral Research and Diagnostic Laboratory (VRDL) at SMS Medical College, Jaipur, Rajasthan, India. This facility serves as a tertiary care referral center.

Study participants and sample collection

The study included patients of all ages and either gender presenting to the outpatient and inpatient departments of SMS Hospital and its attached units with symptoms meeting the case definition for influenza-like illness (ILI). ILI was defined as an acute respiratory infection with measured fever of ≥38°C and cough, with an onset within the last 10 days [12]. Throat swab samples were collected from each participant by trained healthcare personnel. Using a sterile Dacron swab, the posterior pharynx and tonsils were vigorously swabbed. The swab was immediately placed into a vial containing viral transport medium (VTM). Samples were labeled, stored in an icebox at 4°C to maintain the cold chain, and transported to the laboratory for processing on the same day.

Sample processing and nucleic acid extraction

Upon receipt, each sample was assigned a unique identification number. Processing was performed in a Biosafety Level-2 (BSL-2) cabinet with appropriate personal protective equipment. Viral nucleic acid (RNA) was extracted from 400 µL of the VTM sample using the bioMérieux EasyMAG automated extraction system (bioMérieux, France), following the manufacturer's instructions. The extraction was based on the principle of nucleic acid binding to silica in a high-salt buffer. The eluted RNA (100 µL) was used immediately for molecular testing or stored at -80°C.

Molecular detection and typing of influenza viruses

The detection and typing of influenza viruses were performed using a one-step real-time reverse transcription-polymerase chain reaction (rRT-PCR) assay as per the protocols established by the Centers for Disease Control and Prevention (CDC), USA, and the WHO [12].

Briefly, 10 µL of extracted RNA was added to a 15 µL master mix containing primers and probes specific for the universal detection of influenza A virus, influenza B virus, and the human RNase P gene (as an internal control for sample adequacy). Amplification was performed on a real-time PCR system with the following cycling conditions: reverse transcription at 50°C for 20 minutes, initial denaturation at 94°C for 10 minutes, followed by 45 cycles of denaturation at 94°C for 15 seconds, and annealing/extension at 55°C for 30 seconds. A sample was considered positive if the amplification curve for the influenza target crossed the threshold within 40 cycles.

Data analysis

Demographic (age and gender), clinical (symptoms and hospitalization status), and temporal (month of sample collection) data were recorded for all participants. Laboratory results (positive/negative for influenza and virus type) were merged with this dataset. The positivity rate was calculated as the percentage of tested samples that were positive for influenza. Data were entered into Microsoft Excel (Microsoft Corp., Redmond, WA, US) and analyzed using SPSS version 20.0 (IBM Inc., Armonk, New York). Descriptive statistics (frequency and percentage) were used to summarize baseline characteristics. Associations between categorical variables were tested using the Chi-square test, and continuous variables were compared using the Student’s t-test. Statistical significance was set at p<0.05. For all analyses, sample sizes and percentages are presented as n (%).

Ethical considerations

The study was approved by the Institutional Ethics Committee of SMS Medical College, Jaipur (Approval No.: 365/MC/EC/2020). Written informed consent was obtained from all participants or their legal guardians before enrollment and sample collection.

## Results

Overall influenza positivity and type distribution

Over the one-year study period, a total of 7,231 patients presenting with ILI were tested for influenza viruses (Table 1). Among these, 598 (8.26%) were positive (95% CI: 7.63-8.91%). Influenza A virus was the predominant type, detected in 500 cases (6.91% of all tested samples and 83.61% of all positive cases). Influenza B virus was identified in 98 cases (1.35% of all tests and 16.39% of positive cases). The difference in detection rates between influenza A and B was statistically significant (χ²=695.4, p<0.0001).

**Table 1:** Overall detection of influenza viruses among 7,231 patients with influenza-like illness. Comparison of positivity rates between influenza A and B viruses was performed using the Chi-square test.

Virus type	Total tested (N)	Positive cases (n)	Positivity % (95% CI)	% of positive cases	Test statistic	p-value
Influenza A	7,231	500	6.91% (6.33-7.51)	83.61%	χ²=695.4	<0.0001
Influenza B	7,231	98	1.35% (1.09-1.64)	16.39%		
Total	7,231	598	8.26% (7.63-8.91)	100%		

Temporal (monthly) variation in influenza activity

**Figure 1:**
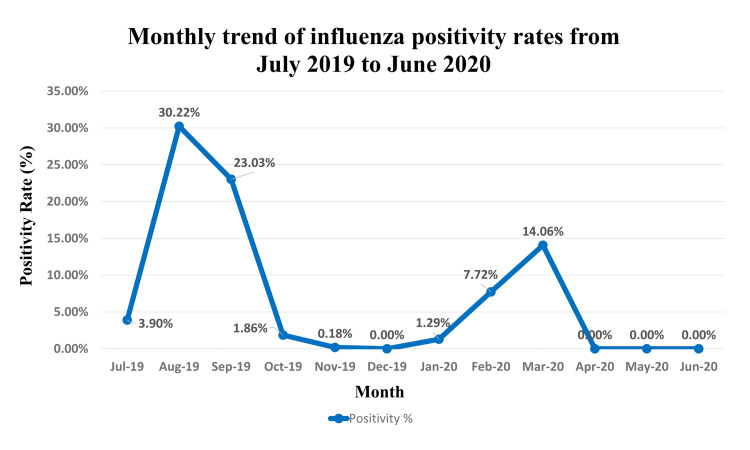
Monthly trend of influenza positivity rates. Positivity rates were calculated from 7,231 patients with influenza-like illness tested between July 2019 and June 2020 in Jaipur, India. The graph shows distinct bimodal peaks during the late monsoon (August-September 2019) and late winter (February-March 2020).

Influenza virus activity demonstrated a clear bimodal seasonal pattern (Table 2). The highest number of positive cases, 216 (36.12%), and a peak in positivity rate (14.06%) occurred in March 2020. A second, more intense peak in the positivity rate was observed in August 2019, 110 cases (30.22%), followed by a high number of cases in September 2019, 193 cases (23.03%). No influenza activity was detected from April to June 2020 or in December 2019. A graphical representation of this monthly trend is provided in Figure 1.

**Table 2:** Monthly distribution of influenza cases and positivity rates from July 2019 to June 2020. The bimodal seasonality is evident from the high positivity rates in August-September 2019 and February-March 2020. No statistical test was applied to these descriptive temporal data.

Month	Total samples	Positive cases (n)	Positivity %
July 2019	205	8	3.90%
August 2019	364	110	30.22%
September 2019	838	193	23.03%
October 2019	700	13	1.86%
November 2019	550	1	0.18%
December 2019	488	0	0.00%
January 2020	620	8	1.29%
February 2020	635	49	7.72%
March 2020	1536	216	14.06%
April 2020	691	0	0.00%
May 2020	455	0	0.00%
June 2020	149	0	0.00%
Total	7231	598	8.26%

Age and gender distribution of positive cases

The distribution of influenza cases across age groups was significantly non-uniform (χ² test for goodness-of-fit, p<0.0001). The highest burden of disease was observed in the 20-40 years age group, which accounted for 233 of the 598 positive cases (38.96%) (Table 3). Analysis by gender revealed a higher number of cases among males, 349 (58.36%), compared to females, 249 (41.63%). This difference was statistically significant (χ²=16.72, p<0.0001), with an odds ratio of 1.95 (95% CI: 1.63-2.33) for males compared to females.

**Table 3:** Distribution of 598 influenza-positive cases across different age groups. The difference in the proportion of cases across age groups was assessed using a Chi-square goodness-of-fit test.

Age group (years)	Positive cases (n)	% of total positive cases	Test statistic	p-value
0-4	84	14.04%	χ²=140.7	<0.0001
5-19	62	10.36%		
20-40	233	38.96%		
41-59	102	17.05%		
>59	117	19.56%		
Total	598	100%		

Clinical presentation and hospitalization status

The clinical presentation of confirmed influenza cases is summarized in Table 4. Fever and cough were universal symptoms in 598 (100%). The other most common symptoms were nasal catarrh in 536 patients (89.63%) and sore throat in 527 patients (88.12%). Shortness of breath was reported in 224 cases (37.45%). A majority of influenza-positive patients were managed on an outpatient basis (375, 62.71%), while 223 patients (37.29%) required hospitalization.

**Table 4:** Clinical profile and hospitalization status of 598 influenza-positive patients. ^a^N/A: Not Applicable, as these are descriptive findings of symptoms present in a confirmed cohort. The difference in hospitalization rates between influenza types (A vs. B) was tested using the Chi-square test.

Parameter	Category	Number (n)	Percentage (%)	Test statistic	p-value
Symptoms	Fever	598	100%	N/A^a^	N/A
	Cough	598	100%		
	Nasal catarrh	536	89.63%		
	Sore throat	527	88.12%		
	Shortness of breath	224	37.45%		
Hospitalization status	Outpatient (OPD)	375	62.71%	χ²=0.54	0.464
	Inpatient (IPD)	223	37.29%		

## Discussion

Our one-year surveillance study identified a clear bimodal pattern of influenza virus activity in North India, with peaks in the late monsoon and late winter, and a predominance of influenza A(H3N2). The key findings include an overall positivity rate of 8.26%, a clear bimodal seasonality with peaks in the late monsoon (August-September 2019) and late winter (March 2020), a predominance of influenza A, and a higher disease burden among young adults aged 20-40 years (233 of 598, 38.96%) and males (349 of 598, 58.36%).

The overall influenza positivity rate of 8.26% observed in our study is lower than the 14.0% reported in a multi-site Indian surveillance study from 2009 to 2013 [10] and the 15.8% reported from Lucknow during 2010-2012 [13]. This lower positivity can likely be attributed to our study’s timing (2019-2020), which coincided with the onset of the COVID-19 pandemic. The implementation of stringent NPIs from April 2020 onward, such as mask mandates, lockdowns, and social distancing, to control SARS-CoV-2 transmission [14], likely contributed to this trend. These measures are known to have concurrently suppressed the circulation of other respiratory viruses, including influenza, a phenomenon reported globally [15]. The WHO also noted historically low influenza activity worldwide during this period, aligning with our findings of zero detections from April to June 2020 [16].

The most significant finding of our study is the distinct bimodal seasonality of influenza, with a primary peak in the late monsoon (August-September) and a secondary, yet substantial, peak in the late winter (February-March). This pattern is consistent with several studies from northern India that have reported twin peaks associated with the monsoon and winter months [17]. For instance, a study from Delhi and other sub-regional sites in India also reported high activity during July-September [10]. This pattern contrasts with the single winter peak observed in temperate countries and highlights the complex interplay of climatic factors such as humidity, temperature, and rainfall in driving influenza transmission in tropical and subtropical regions [18].

Our data revealed that young adults aged 20-40 years constituted the largest proportion, 233 (38.96%), of influenza cases. This is a noteworthy finding, as this demographic is typically the most economically productive and socially mobile, potentially acting as a key vector for community transmission. While influenza-related morbidity and mortality are often higher at the extremes of age, the highest incidence of infection is frequently observed in younger adults [19]. This finding underscores the importance of including this age group in vaccination strategies, not only for their own protection but also to create a buffer to protect more vulnerable populations such as the elderly and those with comorbidities.

We found a significantly higher proportion of influenza cases among males, 349 (58.36%), compared to females. This gender disparity has been observed in other studies from the region [10] and may be influenced by socio-behavioral factors. In the local context, males may have greater exposure due to higher participation in workforce activities outside the home, increased travel, and potentially a higher rate of health-seeking behavior for acute febrile illness compared to females.

As expected, the clinical presentation was classic for influenza, with all confirmed cases presenting with fever and cough. A considerable proportion of patients, 223 (37.29%), required hospitalization, indicating the significant burden of severe influenza disease on the healthcare system. This reinforces the need for robust infection control practices in hospital settings, especially during peak transmission seasons.

Strengths and limitations

A key strength of this study is the large sample size (n=7,231) tested over a full year using a highly sensitive and specific molecular assay (rRT-PCR), providing reliable data on circulating viruses. However, the findings are from a single tertiary care center, which may limit generalizability to the community or other regions of India with different climatic conditions. Furthermore, the study period was impacted by the COVID-19 pandemic, which likely altered the typical epidemiology of influenza in its final months.

## Conclusions

In conclusion, our study confirms a pattern of biannual peaks of influenza activity in Jaipur, Rajasthan, with circulation during the late monsoon and late winter months. Influenza A was the dominant type, with a significant disease burden among young adults (20-40 years), suggesting that they play a crucial role in transmission dynamics.

These findings are critical for shaping local public health responses and emphasize the need for regionally tailored strategies, including concluding vaccination campaigns by July ahead of the monsoon peak and considering a second push in January for the winter peak. Enhanced, continuous surveillance is essential to monitor antigenic drift and novel strains. Furthermore, public health messaging and vaccination efforts should specifically target young and mobile adult populations, who are key vectors for community transmission. Future multi-year, community-based studies will be valuable to further refine our understanding of influenza seasonality in this region and to inform the most effective preventive strategies.
